# Absolute Bioavailability, Tissue Distribution, and Excretion of Erinacine S in *Hericium erinaceus* Mycelia

**DOI:** 10.3390/molecules24081624

**Published:** 2019-04-24

**Authors:** Jun-Hao Hu, I-Chen Li, Ting-Wei Lin, Wan-Ping Chen, Li-Ya Lee, Chin-Chu Chen, Chia-Feng Kuo

**Affiliations:** 1 Department of Food Science, Nutrition, and Nutraceutical Biotechnology, Shih Chien University, Taipei 10462, Taiwan; polo5566789@yahoo.com.tw (J.-H.H.); gkbioeng@grapeking.com.tw (C.-C.C.); 2 Biotech Research Institute, Grape King Bio Ltd., Taoyuan 32542, Taiwan; Ichen.li@grapeking.com.tw (I.-C.L.); tingwei.lin@grapeking.com.tw (T.-W.L.); wp.chen@grapeking.com.tw (W.-P.C.); ly.lee@grapeking.com.tw (L.-Y.L.); 3 Institute of Food Science and Technology, National Taiwan University, Taipei 10617, Taiwan; 4 Department of Bioscience Technology, Chung Yuan Christian University, Taoyuan 32023, Taiwan

**Keywords:** *Hericium erinaceus* mycelia, Erinacine S, bioavailability, tissue distribution, excretion

## Abstract

Erinacine S, so far known to have been produced only in *Hericium erinaceus* mycelia, has just recently been discovered and is able to reduce amyloid plaque growth and improve neurogenesis in aged brain of rats. However, few investigations have been conducted on the absorption, distribution, and excretion study of Erinacine S. This study aimed to investigate the absolute bioavailability, tissue distribution, and excretion of Erinacine S in *H. Erinaceus* mycelia in eight-week old Sprague-Dawley rats. After oral administration and intravenous administration of 2.395 g/kg body weight of the *H. erinaceus* mycelia extract (equivalent to 50 mg/kg body weight Erinacine S) and 5 mg/kg of Erinacine S, respectively, the absolute bioavailability was estimated as 15.13%. In addition, Erinacine S was extensively distributed in organs such as brain, heart, lung, liver, kidney, stomach, small intestine, and large intestine. The maximum concentration of Erinacine S was observed in the stomach, 2 h after the oral administration of *H. erinaceus* mycelia extract, whereas the maximum amount of Erinacine S found in other tissues were seen after 8 h. Total amount of unconverted Erinacine S eliminated in feces and urine in 24 h was 0.1% of the oral dosage administrated. This study is the first to show that Erinacine S can penetrate the blood–brain barrier of rats and thus support the development of *H. erinaceus* mycelia, for the treatment of neurological diseases.

## 1. Introduction

*Hericium erinaceus*, also known as the lion’s mane mushroom, *hou tou gu*, or *yamabushitake*, is an edible mushroom with medicinal values that has a long history of usage in Eastern Asia [1]. This mushroom has been reported to possess anti-cancer [2], immuno-modulating [3], anti-hyperglycemic [4], anti-hyperlipidemic [4], anti-oxidant [5], anti-osteoporotic [5], anti-bacterial [6], wound healing [7], anti-neurodegenerative [8], neurogenesis-inducing, and memory-improving activities [9,10,11]. Moreover, *H. erinaceus* has been shown to prevent ischemia injury [12], Alzheimer’s disease [13], Parkinson’s disease [14], and depression [15] in animal models. Hence, this mushroom has attracted attention for clinical experiments and development of functional foods, due to its potential health benefits.

Erinacine S, a novel sesterterpene in the mycelia of *Hericium erinaceus*, has recently been discovered and was found to have the ability to attenuate Aβ plaque burden in the brains of five-month-old female APP/PS1 transgenic mice [16]. The inhibition of glial cells and enhancement of insulin degrading enzyme expression were shown to be the underlying mechanisms for Erinacine S in reducing amyloid plaque growth and improving neurogenesis, respectively, in the aged brain [17].

Despite the fact that many phytochemicals have the capabilities to elicit various physiological effects in vitro, many of them failed to extrapolate the results of these studies in humans [18,19]. The most common reason for the clinical failure was the instability in the gut and the poor bioavailability of the bioactive compound [20]. Therefore, preclinical absorption, distribution, and excretion screening of the compound should be investigated as early as possible to eliminate weak candidates. Currently, there has been an analytical method reported for determining Erinacine S contained in the crude herb [16]. However, this method has not been used in the pharmacokinetic study of Erinacine S in biological samples. Hence, this is the first study to address the preclinical pharmacokinetics of Erinacine S after oral administration of *H. erinaceus* mycelia. It was expected that the results of this study will provide a basis for elucidating the pharmacodynamic effects of Erinacine S and the clinical use of *H. erinaceus* mycelia.

## 2. Results

### 2.1. Method Validation

**Figure 1:**
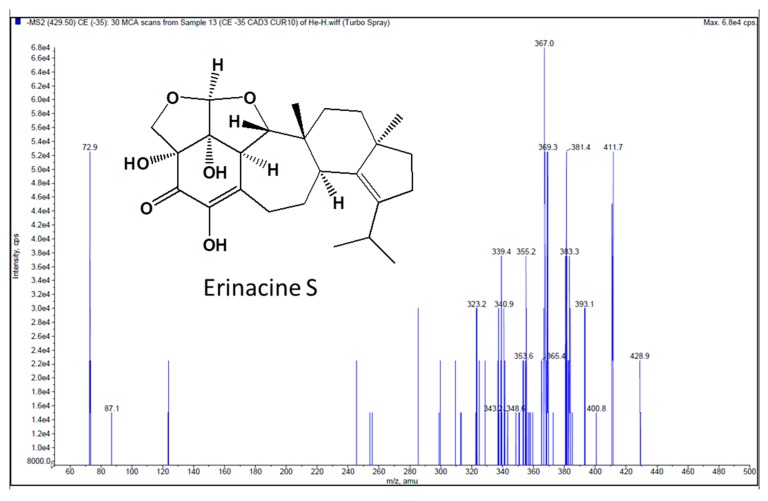
Product ion spectrum of Erinacine S.

**Figure 2:**
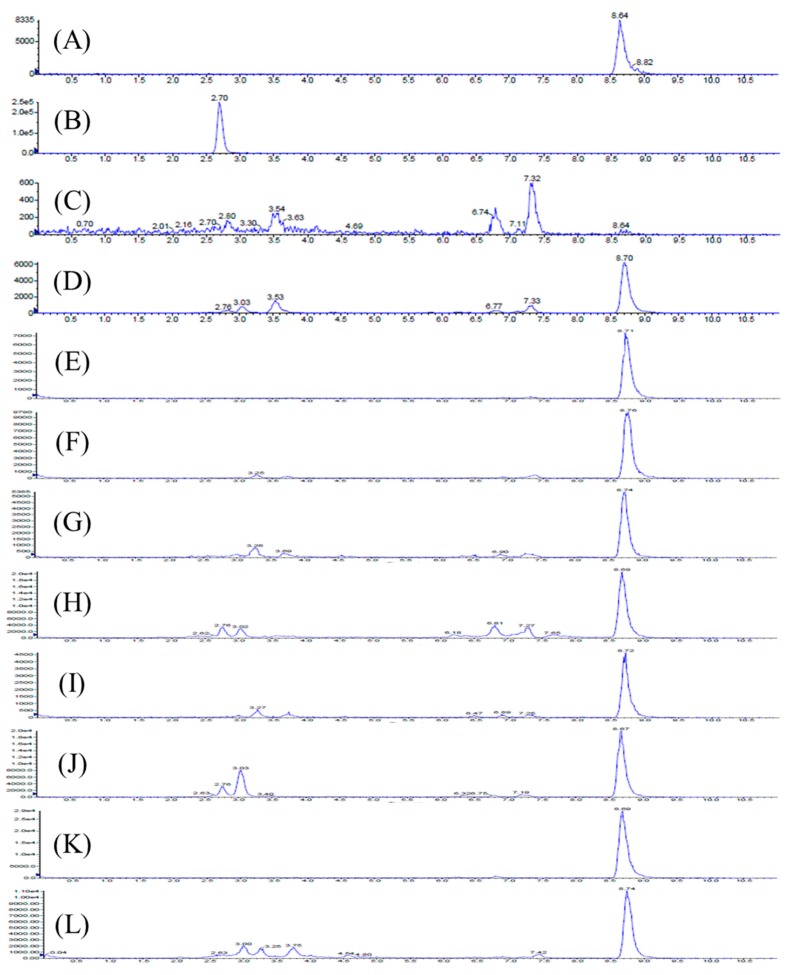
Representative HPLC Chromatograms for (**A**) Erinacine S (100 ng/mL); (**B**) internal standard (4-hydroxybenzaldehyde; 50 ng/mL); (**C**) blank rat plasma; (**D**) rat plasma sample obtained 360 min after a 2.395 g/kg body weight oral dose of *Hericium erinaceus* mycelia extract (equivalent to 50 mg/kg body weight of Erinacine S); (**E**) brain, (**F**) heart, (**G**) lung, (**H**) liver, (**I**) kidney, (**J**) stomach, (**K**) small intestine, and (**L**) large intestine samples obtained 8 h after a 2.395 g/kg body weight oral dose of the *H. erinaceus* mycelia extract.

The product ion spectrum of Erinacine S and the typical Multiple reaction monitoring (MRM) chromatograms of rat plasma and tissue samples after administration of Erinacine S are shown in Figure 1 and Figure 2, respectively. Retention times for Erinacine S and internal standard 4-hydroxybenzaldehyde were detected at 8.7 min and 2.70 min, respectively. No significant interference from endogenous materials was observed in the rat plasma or the tissue homogenates at the retention time of Erinacine S or the internal standard. Therefore, the assay condition provided an adequate specificity to characterize the pharmacokinetics and tissue distribution of Erinacine S.

The intra-day and inter-day precision and accuracy for Erinacine S after analyzing six replicates of the nine different concentrations are shown in Table 1. The precision and accuracy for analyses at 5–500 ng/mL Erinacine S were found to be within the acceptable limits (≤15%) [21]. The results also showed a linear fit from 5 to 500 ng/mL with the relationship of y = 1.00x + 1.19 and a regression coefficient of 0.999.

The extraction recoveries of Erinacine S in rat plasma and the tissues are shown in Table 2. The extraction recoveries of Erinacine S were higher than 97% and 90% for the plasma and for most tissues, respectively.

### 2.2. Pharmacokinetic Study

**Figure 3:**
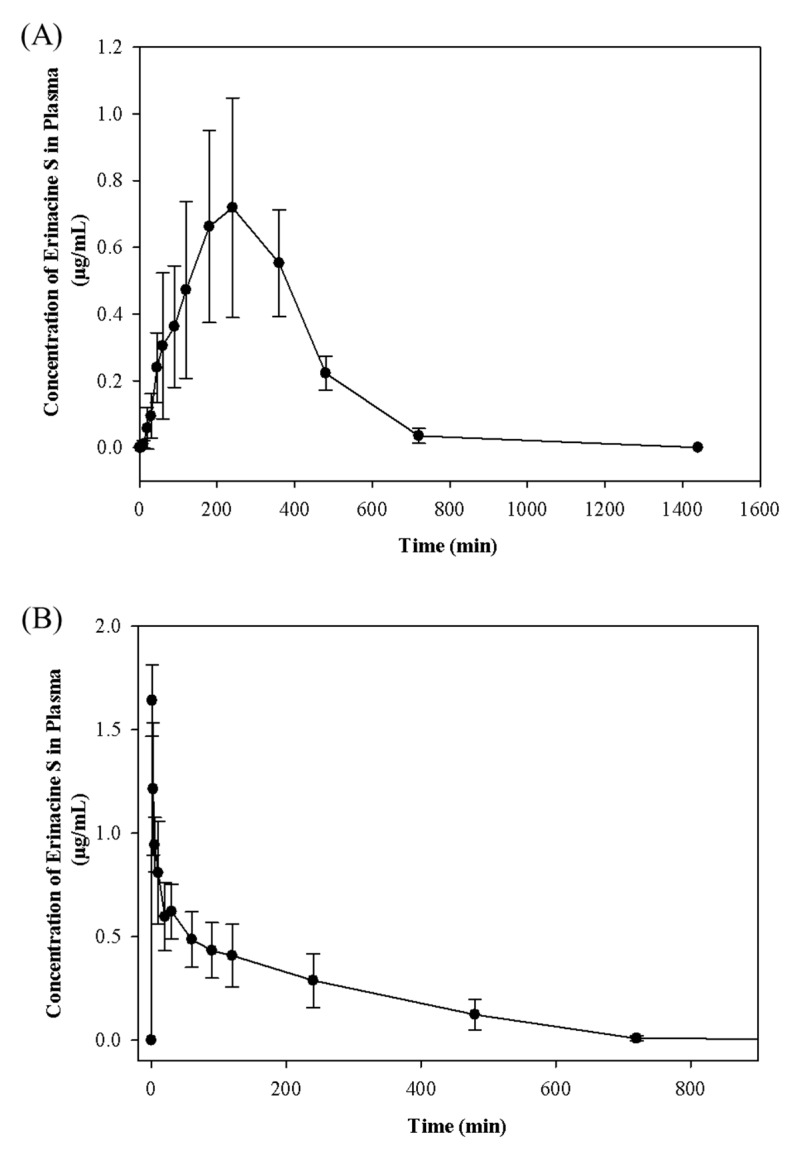
Plasma concentration–time curves of Erinacine S in rats after (**A**) oral administration of *H. erinaceus* mycelia extract at 2.395 g/kg body weight (equivalent to 50 mg/kg body weight of Erinacine S) and (**B**) intravenous administration of Erinacine S at 5 mg/kg. Values are means ± SD (*n* = 6).

The developed and validated LC-MS/MS method was successfully applied to monitor the time course of Erinacine S after oral administration of 2.395 g/kg body weight *H. erinaceus* mycelia extract (equivalent to 50 mg/kg body weight of Erinacine S) or intravenous injection of 5 mg/kg Erinacine S. The plasma concentration-time profiles after oral and intravenous administrations are presented in Figure 3 and the mean pharmacokinetic parameters are summarized in Table 3. After the oral administration of *H. erinaceus* mycelia extract, the plasma concentration of Erinacine S increased sharply, and reached a peak concentration (C_max_) of 0.73 ± 0.31 μg/mL at 270.00 ± 73.48 min. When the peak concentration was reached, Erinacine S with a half-life (T_1/2_) of 439.84 ± 60.98 min, was eliminated. For intravenous administrations, the maximum plasma concentration of Erinacine S was 1.64 ± 0.17 μg/mL, with a half-life (T_1/2_) of 11.45 ± 5.76 min. The area under the curve (AUC) values for the plasma concentration-time curve of the oral and intravenous administrations of Erinacine S were 272.06 ± 77.82 and 179.77 ± 66.46 min μg/mL, respectively. The absolute oral bioavailability of Erinacine S in rats was estimated to be 15.13%.

### 2.3. Tissue Distribution Study

**Figure 4:**
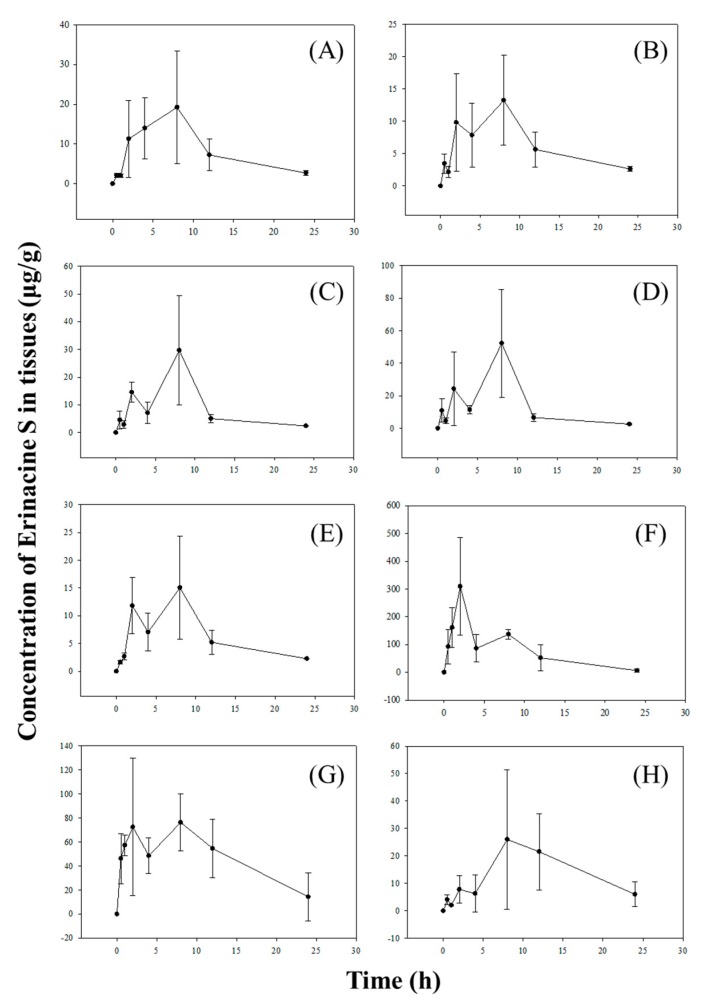
Mean plasma concentration–time curves of Erinacine S in (**A**) brain, (**B**) heart, (**C**) lung, (**D**) liver, (**E**) kidney, (**F**) stomach, (**G**) small intestine, and (**H**) large intestine of rats, after oral administration of the *H. erinaceus* mycelia extract at 2.395 g/kg body weight (equivalent to 50 mg/kg body weight of Erinacine S). Values are means ± SD (*n* = 6).

The tissue distributions of Erinacine S were determined within 24 h after oral administration of the *H. erinaceus* mycelia extract at 2.395 g/kg body weight (equivalent to 50 mg/kg body weight of Erinacine S). As shown in Figure 4, Erinacine S was widely distributed in all of the main organs, including the brain, heart, lung, liver, kidney, stomach, small intestine, and the large intestine; the highest concentrations of Erinacine S were detected in the stomach at 2 h after administration (309.672 ± 175.731 μg/g). Erinacine S was detected in the brain, as early as half hour after administration (2.069 ± 0.503 μg/g), peaked at 2 h after administration (11.294 ± 9.662 μg/g), and reached the maximum at 8 h (19.238 ± 14.239 μg/g). This showed that Erinacine S could penetrate the blood–brain barrier of rats.

The concentration of Erinacine S had also peaked in the heart (9.816 ± 7.500 μg/g), lung (14.549 ± 3.564 μg/g), liver (24.309 ± 22.544 μg/g), kidney (11.800 ± 5.061 μg/g), the small intestine (72.483 ± 57.190), and the large intestine (7.784 ± 5.057 μg/g), 2 h after administration, and reached the maximum at 8 h (13.246 ± 6.957, 29.710 ± 19.815, 52.352 ± 33.099, 15.053 ± 9.317, 76.401 ± 23.772, and 25.965 ± 25.384 μg/g, respectively).

### 2.4. Excretion Study

The excretion of Erinacine S in urine and feces, after oral administration of the *H. erinaceus* mycelia extract, are shown in Table 4. The fecal excretion of the unmetabolized form of Erinacine S gradually increased with time, from 0.273 ± 0.236 μg/mL at 0–4 h, 3.482 ± 4.401 μg/mL at 4–8 h, 9.724 ± 3.036 μg/mL at 8–12 h, to 9.546 ± 3.182 μg/mL in the 12–24 h period, and the cumulative amount was 0.094 ± 0.053% of the administered dose. Meanwhile, the urinary excretion of the unmetabolized form of Erinacine S remained unchanged at different intervals and the cumulative excreted fraction was only 0.004 ± 0.002%.

## 3. Discussion

This was the first report on the pharmacokinetic studies of Erinacine S in biological samples, following the oral administration of the *H. erinaceus* mycelia extract and intravenous injection of Erinacine S. According to earlier assessments of acute toxicity, genotoxicity, subchronic toxicity, and developmental toxicity studies of *H. erinaceus* mycelia, no observable adverse effects were found [22,23,24]. Consistent with those of previous reports, no toxicity signs were observed when 2.395 g/kg body weight of the *H. erinaceus* mycelia extract or 5 mg/kg body weight Erinacine S was given to the rats in this study.

Since this was the first bioavailability study for Erinacine S, the appropriate dose was selected on the basis of our preliminary results. The preliminary data showed that 1 g of freeze-dried *H. erinaceus* mycelia powder could only yield 4.2 mg of Erinacine S. If the rats were to be given an equivalent 50 mg/kg body weight of Erinacine S by oral gavage, they would have to be exposed to 11.905 g/kg body weight of the freeze-dried *H. erinaceus* mycelia powder, which would exceed the maximal administration volumes (1 mL/l00 g body weight) that can be administered [25]. In addition, Erinacine S concentrations in the plasma of rats exposed to pure Erinacine S was undetectable after 240 min and had an oral bioavailability of only 1.3%. It might be that Erinacine S was destroyed by gastric acid or got metabolized, resulting in poor bioavailability [26,27]. Hence, for the best resolution, *H. erinaceus* mycelia extract instead of pure Erinacine S was chosen for oral feeding in this study.

Erinacine S was rapidly detected at the first blood sampling time point (1 min) and a double-peak phenomenon was observed in the plasma profile, 30 min after intravenous administration of Erinacine S. However, only a single peak was exhibited in the plasma profile after oral administration of the *H. erinaceus* mycelia extract. The main explanations for the double-peak phenomenon have been the effects of enterohepatic circulation [28], delayed gastric emptying [29], or variability of gastrointestinal absorption [30]. Since Erinacine S was injected directly into the bloodstream, it would be unlikely that delayed gastric emptying or variability of absorption could be the major reason for the double peak. Therefore, it is most likely that Erinacine S underwent enterohepatic circulation in rats. Erinacine S could enter the portal circulation, go to the liver, be excreted into the bile, get passed on to the intestinal lumen, get reabsorbed across the intestinal mucosa, and be returned to the liver via portal circulation, which explained why a second peak was seen after 30 min. Although similar double-peak phenomenon has been observed in the pharmacokinetic studies of other compounds, including xanthohumol [31] or luteolin [32], further research is needed to confirm the pathways of the enterohepatic circulation of Erinacine S.

Other than the brain, the double-peak phenomenon was also detected in many tissues such as heart, liver, lung, kidney, stomach, small intestine, and large intestine. The two peaks were approximately 6 h apart. The maximum concentration of Erinacine S was observed in the stomach 2 h, following oral administration of the *H. erinaceus* mycelia extract, whereas the maximum amount of Erinacine S found in other tissues were seen after 8 h. At 2 h, after oral dosing, the maximum Erinacine S concentrations in the stomach (309.672 μg/g) were 4.27 times higher than that in small intestines (72.483 μg/g), indicating that most of the Erinacine S in the stomach was absorbed across the gastric epithelium, before being delivered into the small intestine. Besides the enterohepatic circulation, the delay in stomach emptying might also have contributed towards the establishment of the double-peak phenomenon, since more than 50% of Erinacine S took more than 2.5 h to reach the small intestine [33].

The permeability from the systemic circulation to various tissues depended largely on the physicochemical properties of the drug [34]. Considering Erinacine S is hydrophobic and insoluble in water, it might ahve penetrated the cell membranes more easily, when compared with drugs that were more water soluble and polar. Results showed that Erinacine S was not only detectable in various tissues, but also in the brain, 30 min after oral administration. The concentration of Erinacine S increased over time and reached the maximum of 19.238 μg/g at 8 h. Unlike other peripheral tissues that allowed a free exchange of metabolites between the blood and tissues, the blood–brain barrier in the brain is formed by tight junctions and surrounded by glial cells that limit the penetration of molecules into the brain [35]. Hence, this study demonstrated for the first time ever that Erinacine S could cross the blood brain barrier and exert its beneficial effect for the treatment of neurological disorders, such as Alzheimer’s disease (AD) [17].

The intake and distribution of the drug should be balanced with the elimination so that therapeutic goal can be achieved with minimal adverse effects. However, as Erinacine S is involved in an enterohepatic circulation, its excretion might last longer than expected. Erinacine S in urine samples reached a peak at 0–8 h, gradually decreased to a concentration of 0.068 ± 0.038 μg/mL at 8–12 h, and reached a peak again at 12–24 h, while the highest concentration of Erinacine S in fecal samples was found between 8–24 h. As Erinacine S was measured in the urine and fecal samples within 24 h after oral administration, more unchanged Erinacine S could possibly be detected after 24 h. The total amount of Erinacine S collected in urine and feces for 24 h were 0.004% and 0.094% of oral dosage, respectively. Although the feces appeared to be the major route of elimination, the total amount of unchanged Erinacine S found in the urine and fecal samples was less than 0.1%, implying that approximately 99.9% of Erinacine S should either be absorbed or metabolized into other molecules [36].

## 4. Materials and Methods

### 4.1. Preparation of H. erinaceus Mycelia

*H. erinaceus* obtained from the Bioresources Collection and Research Center in Food Industry Research and Development Institute (BCRC 35669; Hsinchu, Taiwan) was cultured on potato dextrose agar at 26 °C for 15 days. After incubation, the fungal mycelia were cut from the agar and grown in 2 L flasks containing 1.3 L of synthetic medium (0.25% yeast extract, 4.5% glucose, 0.5% soybean powder, 0.25% peptone, and 0.05% MgSO_4_, adjusted to pH 4.5) on a rotary shaker incubator at 120 rev/min at 25 °C for 5 days. Then the shake flasks were scaled up to 500 L fermenters and 20 ton fermenters for 5 days and 12 days, respectively. After cultivation, the mycelia were harvested, lyophilized, grounded, and stored in a desiccator, at room temperature.

### 4.2. Preparation of the H. erinaceus Mycelia Extract and Erinacine S

Ethanol extract of the Erinacine S from *H. erinaceus* mycelia was prepared according to a published paper [17]. Briefly, freeze-dried *H. erinaceus* powder was mixed with 95% ethanol, sonicated for 2 h, and filtered through Whatman No. 1 paper, by vacuum filtration. The supernatant was then concentrated by use of a rotary evaporator to obtain the *H. erinaceus* mycelia extract. Then, the extract was suspended in H_2_O, successively partitioned with ethyl acetate (EtOAc), and fractionated over a silica gel column (70–230 mesh, 70 × 10 cm) using a gradient system of *n*-hexane/EtOAc (10:1; 3:1; 3:2; 1:1; 1:2; 0:1) to provide seven fractions (Fr.I-VII). Fraction III, the elute of *n*-hexane/EtOAc (3:2), was further chromatographed on Sephadex LH-20 and silica gel columns to afford Erinacine S.

### 4.3. Bioanalysis of Erinacine S

Erinacine S concentration in the samples were analyzed using Agilent 1100 series LC system equipped with a G1376A capillary pump and a G1313A autosampler, according to a previous published method [16]. Chromatographic separation was performed on an Agilent Eclipse XDB-C18 column (3.5 µm, 4.6 × 100 mm), using a mobile phase consisting of water (A) and acetonitrile (B), at a flow rate of 0.35 mL/min. The injection volume was 10 μL and the gradient elution program was as follows—0 min, 70% B; 0–5 min, 70–100% B; 5–8 min, 100% B; 8–8.1 min, 100–70% B; and 8.1–11 min 70% B. Mass spectrometric detection was carried out on a triple quadrupole mass spectrometer (API 3000; Applied Biosystems, Vaughan, Ontario, Canada), using Turbo Ion spray as source, coupled with electro spray ionization interfaces, operating in a negative ion mode and ion spray voltage at −4500 V. The source turbo spray consisted of a nebulizer gas at 10 psi, collision gas at 2 psi, curtain gas at 7 psi, with a source temperature at 275 °C. Multiple reaction monitoring (MRM) mode was employed for the quantification—*m/z* 429.3 → 411.3 for Erinacine S (rt = 8.7 min), and *m/z* 121 → 91.9 for 4-hydroxybenzaldehyde (internal standard). Data processing was performed with the Analyst 1.4.2 software (Applied Biosystems, Concord, ON, Canada).

### 4.4. Method Validation

The method was validated according to the guidelines set by the European Medicines Agency [37]. The specificity of the method was evaluated by comparing the chromatograms of blank rat plasma samples (spiked with Erinacine S and internal standard 4-hydroxybenzaldehyde) with the actual plasma and tissue samples obtained after oral administration of the *H. erinaceus* mycelia extract.

Intra- and inter-day variations were carried out by analyzing Erinacine S at 7 concentration levels (5, 10, 20, 50, 100, 200, and 500 ng/mL) in six replicate samples, on three consecutive validation days, as follows. Precision and accuracy were expressed as percentage coefficient of variation (% CV) and percentage bias (% bias) of the replicate measurements, respectively. Percentage CV was calculated as (standard deviation/mean) × 100, while % bias was calculated as [(measured concentration—theoretical concentration)/theoretical concentration] × 100. The methods are acceptable when the precision and accuracy of the intra-day and inter-day are within ±15% [21].

Recoveries were assessed by comparing the peak areas of the extracted samples (spiked before extraction) to that of the unextracted samples (spiked after extraction). Briefly, 40 μL of Erinacine S at the three different concentrations (1, 4, 10 μg/mL) were added to 160 μL of plasma or tissue homogenates. After this, 100 μL of internal standard 4-hydroxybenzaldehyde (final concentration 25 ng/mL) and 500 μL ethyl acetate was added to the solution, followed by vortexing for 1 min and centrifuging at 13,845 g for 3 min. The supernatant was evaporated to dryness and the residue was reconstituted with isovolumetric acetonitrile for LC-MS/MS analysis. Extraction recovery (%) was calculated as—(measured concentration/theoretical concentration) × 100% [38].

### 4.5. Animal Study

Eight-week-old male Sprague-Dawley rats purchased from BioLASCO Co (Taipei, Taiwan) were maintained in environmentally controlled rooms (22 ± 2 °C; 40–60% humidity), with diurnal lighting on a 12:12 h light–dark cycle. The chow diet (MF 18 Rodent diet, Oriental Yeast Co., Tokyo, Japan) and water were given ad libitum. All animal procedures were approved by the Animal Care and Use Committee of the Shih Chien University and were carried out in accordance with the National Institutes of Health guidance for the care and the use of laboratory animals.

### 4.6. Pharmacokinetic Study

For the pharmacokinetic study, twelve rats were randomly assigned to two groups (*n* = 6) and orally administrated with the *H. erinaceus* mycelia extract (2.395 g/kg body weight; equivalent to 50 mg/kg body weight Erinacine S) or intravenous administrated with Erinacine S dissolved in dimethyl sulfoxide (DMSO) (5 mg/kg). Before intravenous administration, animals were anesthetized with Avertin (2,2,2-Tribromoethanol, 0.071 M). Blood was collected at scheduled time points (0, 5, 10, 20, 30, 45, 60, 90, 120, 180, 240, 360, 480, 720, and 1,440 min post-dose for the *H. erinaceus* mycelia extract and 0, 1, 2.5, 5, 10, 20, 30, 60, 90, 120, 240, 480, 720, and 1,440 min post-dose for Erinacine S) into heparinized microtubes and centrifuged at 2,404 g for 10 min. The plasma (100 μL) was then mixed with equal volumes of the internal standard 4-hydroxybenzaldehyde (final concentration 25 ng/mL) and 300 μL of ethyl acetate. After the mixing, the mixture was vortexed for 1 min and centrifuged at 13,845 g for 3 min. The supernatant was evaporated to dryness and the residue was reconstituted with isovolumetric acetonitrile for LC-MS/MS analysis [38].

### 4.7. Distribution Study

For the tissue distribution study, the rats received a single oral administration of the *H. erinaceus* mycelia extract at 2.395 g/kg and were sacrificed at 0, 0.5, 1, 2, 4, 8, 12, or 24 h, after administration (*n* = 3 at each time point). Brain, heart, liver, lung, kidney, and gastrointestinal tract (chyme was removed before the wash) were collected and washed by 0.9% NaCl, before being weighted and homogenized in four volumes of saline. The homogenates (100 μL) were then mixed with equal volumes of the internal standard 4-hydroxybenzaldehyde (final concentration 25 ng/mL) and 300 μL of ethyl acetate. After the mixing, the mixture was vortexed for 1 min and centrifuged at 13,845 g for 3 min. The supernatant was evaporated to dryness and the residue was reconstituted with isovolumetric acetonitrile for the LC-MS/MS analysis [38].

### 4.8. Excretion Study

For the excretion study, feces and urine samples were collected at 0–4, 4–8, 8–12, and 12–24 h (*n* = 6), after the rats received a single oral administration of the *H. erinaceus* mycelia extract at 2.395 g/kg. Feces were weighted, freeze-dried, and grinded, before mixing with an equal volume of the internal standard 4-hydroxybenzaldehyde (final concentration 25 ng/mL) and 300 μL of ethyl acetate. Urines were mixed with equal volumes of the internal standard 4-hydroxybenzaldehyde (final concentration 25 ng/mL) and 300 μL of ethyl acetate. After the mixing, the mixture was vortexed for 1 min, centrifuged at 13,845g for 3 min. The supernatant was evaporated to dryness and the residue was reconstituted with an isovolumetric acetonitrile for the LC-MS/MS analysis [38].

### 4.9. Data Analysis

All results were expressed as arithmetic mean ± standard deviation (SD). Maximal plasma concentration (C_max_), and time taken to achieve the maximal plasma concentration (T_max_) were taken directly from the observed data. The area under the plasma concentration time curve (AUC) were performed on each individual animal, using the software of WinNonlin (Pharsight Corp., Mountain View, CA, USA) by the non-compartmental model. The elimination half-life (T_1/2_) was determined by the equation—T_1/2_ = 0.693/λ, where λ was determined by a linear regression based on the terminal phase of plasma concentration. Using the pharmacokinetic data of oral administration (po) and intravenous administration (iv), the absolute bioavailability of Erinacine S was calculated as [(AUC_po_ × Dose_iv_)/(AUC_iv_ × Dose_po_)] × 100% [39].

## 5. Conclusions

Pharmacokinetics of Erinacine S after oral dosing at 2.395 g/kg BW of the *H. erinaceus* mycelia extract (equivalent to 50 mg/kg body weight of Erinacine S) in male Sprague-Dawley rats was 15.13%. The stomach was the main site of Erinacine S absorption, while the fecal excretion was the major route of elimination of the Erinacine S. This study was the first to show that Erinacine S could penetrate the blood–brain barrier of rats and support the development of *H. erinaceus* mycelia for the treatment of neurological diseases.

## Figures and Tables

**Table 1:** Intra-day and inter-day precision and accuracy of LC-MS/MS method for the determination of Erinacine S.

	Intra-Day			Inter-Day		
Theoretical Conc.	Observed Conc.	Precision	Accuracy	Observed Conc.	Precision	Accuracy
(ng/mL)	(ng/mL)	(% CV)	(% bias)	(ng/mL)	(% CV)	(% bias)
5	5.69 ± 0.47	8.29	13.83	5.70 ± 0.44	7.69	14.04
10	8.71 ± 0.88	10.14	−12.94	9.13 ± 0.63	6.88	−8.69
20	20.62 ± 1.62	7.86	3.12	19.36 ± 0.43	2.23	−3.18
50	49.96 ± 1.55	3.1	−0.08	45.31 ± 1.22	2.69	−9.38
100	111.40 ± 1.32	1.18	11.4	97.91 ± 5.09	5.2	−2.09
200	198.56 ± 5.22	2.63	−0.72	206.33 ± 2.54	1.23	3.17
500	499.83 ± 9.19	1.84	−0.03	499.65 ± 6.62	1.33	−0.07

Data expressed as means ± SD (*n* = 6). CV (%) = (standard deviation/ mean) × 100%. bias (%) = [(measured concentration − theoretical concentration)/theoretical concentration] × 100%.

**Table 2:** Extract recoveries (%) of Erinacine S in rat plasma and tissues.

	Theoretical Conc. (ng/mL)		
	50	200	500
Plasma	99.29 ± 4.70	97.63 ± 4.66	100.40 ± 2.37
Brain	102.53 ± 4.47	98.12 ± 2.69	95.98 ± 4.57
Heart	99.54 ± 4.09	83.04 ± 5.96	91.73 ± 5.96
Liver	92.18 ± 9.60	80.46 ± 3.97	91.66 ± 10.15
Lung	100.40 ± 11.72	94.44 ±9.56	98.48 ± 6.25
Kidney	101.69 ± 4.14	98.48 ± 5.35	87.94 ± 4.49
Stomach	77.05 ± 5.96	86.84 ± 5.80	90.33 ± 4.15
Small Intestine	81.78 ± 11.74	85.77 ± 7.12	89.88 ± 5.97
Large Intestine	90.67 ± 11.24	93.63 ± 10.12	101.04 ± 6.64
Feces	101.15 ± 11.12	97.03 ± 9.54	96.78 ± 3.98
Urine	100.87 ± 1.80	101.58 ± 3.71	99.43 ± 3.31

Data expressed as means ± SD (*n* = 6). Recovery (%) = (measured concentration/theoretical concentration) × 100%.

**Table 3:** Pharmacokinetic parameters of Erinacine S in rat plasma after oral administration of *H. erinaceus* mycelia extract at 2.395 g/kg body weight (equivalent to 50 mg/kg body weight of Erinacine S) and intravenous administration of Erinacine S at 5 mg/kg.

	P.O.	I.V.
	(50 mg/kg)	(5 mg/kg)
T_max_ (min)	270.00 ± 73.48	-
C_max_ (μg/mL)	0.73 ± 0.31	1.64 ± 0.17
T_1/2_ (min)	439.84 ± 60.98	11.45 ± 5.76
AUC (min μg/mL)	272.06 ±77.82	179.77 ± 66.46
Absolute Bioavailability (%)	15.13	

Data expressed as mean ± SD (*n* = 6). T_max_—the time taken to reach the maximum concentration. C_max_—maximum plasma concentration. T_1/2_—half-life. AUC—area under the plasma concentration-time curve. Absolute bioavailability (%) = [(AUCpo × Doseiv)/(AUCiv × Dosepo)] × 100%. po—oral administration; iv—intravenous administration.

**Table 4:** Fecal and urinary excretion of Erinacine S after oral administration of the *H. erinaceus* mycelia extract at 2.395 g/kg body weight (equivalent to 50 mg/kg body weight of Erinacine S).

Time (h)	Feces	Urine
	Concentration (μg/kg)	Concentration (μg/kg)
0–4	0.273 ± 0.236	0.094 ± 0.055
4–8	3.482 ± 4.401	0.094 ± 0.062
8–12	9.724 ± 3.036	0.068 ± 0.038
12–24	9.546 ± 3.182	0.086 ± 0.049
Total Amount (μg) (% of administered dose)	21.669 ± 11.479 (0.094 ± 0.053%)	0.932 ± 0.602 (0.004 ± 0.002%)

Data expressed as means ± SD (*n* = 6).
